# Hypovolemic Shock after Circumcision

**Published:** 2014-04-01

**Authors:** Sevgi Buyukbese Sarsu, Burcu Belen

**Affiliations:** Pediatric Surgery Department, Gaziantep Children’s Hospital, Gaziantep, TURKEY; Pediatric Hematology and Oncology Department, Gaziantep Children’s Hospital, Gaziantep, TURKEY

**Dear Sir,**

Circumcision has been the most frequently applied surgical procedure since ancient times [1,2]. In Turkey, the application of this surgical procedure continues due to traditional and religious reasons. Iron deficiency anemia (IDA) is a common pediatric problem affecting up to 25% of all the children worldwide [3].Even though circumcision is known to be a procedure with minor complications, it could cause hypovolemic shock especially in patients with underlying IDA.

A 2.5-month-old infant with hypovolemic shock was brought with bleeding 12 hours after circumcision by a traditional circumciser at home. He was lethargic with pale skin, mucous membranes, and had shallow breathing. The pulse was 180/ minute and arterial blood pressure was not measurable. He had bleeding from the frenulum between two sutures. The bleeding could not be ceased despite compression. The patient was given 20cc/kg of Ringer’s lactate solution. Laboratory tests revealed Hb 4.6g/dl; hematocrit 14.6%; and his mean corpuscular volume (MCV) was 48fl, total iron 7 g/l , serum ferritin 9 ng/ml with normal PT, APTT, fibrinogen and platelet levels. He was transfused with packed red blood cells. The urine density of the patient was 1040 following transfusion. The bleeding was stopped following the suturing of the frenulum. 

It has been widely reported that circumcision complications occur more frequently with increasing age of the patient. The early complications of circumcision are bleeding, pain, inadequate skin removal, and surgical site infection, but they usually tend to be minor. Of these, bleeding is the most common complication of circumcision. Bleeding may occur along the skin edges between sutures or from a discrete blood vessel, most commonly at the frenulum. In our patient, the bleeding was from the frenulum. The majority of post-circumcision bleeding would not require wound exploration and suturing, and responds well to compression. However, the application of direct pressure in our case did not cease the bleeding. Bleeding from skin edges usually respond well to compression alone. In Turkey and other developing countries, traditional circumcisers still exist [4]. Our center is a regional institution that mostly serves patients with low socio-economic status. Only a minor percentage of circumcisions in this region are performed by pediatric surgeons. However, a big percentage of post-circumcision complications are treated by pediatric surgeons. Our patient did not have coagulation disorder. The hypovolemic shock which developed after the circumcision was further compounded by patient’s existing IDA. We recommend circumcision should be performed by trained personnel. Moreover, anemia should be treated before performing circumcision especially in endemic areas of IDA. 

## Footnotes

**Source of Support:** Nil

**Conflict of Interest:** None declared

